# Parallel effects of processing fluency and positive affect on familiarity-based recognition decisions for faces

**DOI:** 10.3389/fpsyg.2014.00328

**Published:** 2014-04-22

**Authors:** Devin Duke, Chris M. Fiacconi, Stefan Köhler

**Affiliations:** Department of Psychology, Brain and Mind Institute, Western UniversityLondon, ON, Canada

**Keywords:** recognition memory, masked priming, heuristics, dual-process model, discrepancy attribution

## Abstract

According to attribution models of familiarity assessment, people can use a heuristic in recognition-memory decisions, in which they attribute the subjective ease of processing of a memory probe to a prior encounter with the stimulus in question. Research in social cognition suggests that experienced positive affect may be the proximal cue that signals fluency in various experimental contexts. In the present study, we compared the effects of positive affect and fluency on recognition-memory judgments for faces with neutral emotional expression. We predicted that if positive affect is indeed the critical cue that signals processing fluency at retrieval, then its manipulation should produce effects that closely mirror those produced by manipulations of processing fluency. In two experiments, we employed a masked-priming procedure in combination with a Remember-Know (RK) paradigm that aimed to separate familiarity- from recollection-based memory decisions. In addition, participants performed a prime-discrimination task that allowed us to take inter-individual differences in prime awareness into account. We found highly similar effects of our priming manipulations of processing fluency and of positive affect. In both cases, the critical effect was specific to familiarity-based recognition responses. Moreover, in both experiments it was reflected in a shift toward a more liberal response bias, rather than in changed discrimination. Finally, in both experiments, the effect was found to be related to prime awareness; it was present only in participants who reported a lack of such awareness on the prime-discrimination task. These findings add to a growing body of evidence that points not only to a role of fluency, but also of positive affect in familiarity assessment. As such they are consistent with the idea that fluency itself may be hedonically marked.

## Introduction

Most people can relate to experiencing an immediate and compelling sense of familiarity toward an individual, despite being unable to recall a specific past encounter with that person. This familiarity experience was illustrated by Mandler in his classic butcher-on-the-bus scenario: One may encounter the town butcher in an unusual context, i.e., on the bus, and be left with an impression of familiarity in the absence of successful recovery of any episodic context of a prior meeting (Mandler, 1980). This sense of *familiarity* can be contrasted with a recollective recognition experience, in which contextual details of a specific past encounter can be recovered. In dual-process models of recognition memory, these two recognition experiences have been linked to two distinct retrieval processes, i.e., familiarity assessment and recollection (see Yonelinas, 2002, for review). In the current paper, we aim to shed light on the cognitive mechanisms that underlie familiarity-based recognition memory decisions for faces, with a particular focus on the role of fluency and positive affect.

Dating back to research in the early 1980s, there have been suggestions in the literature that impressions of familiarity may not necessarily be the result of direct access to a pertinent stored representation, but can be based on other, more indirect sources. One such source that has undergone considerable investigation in cognitive research on recognition memory is *processing fluency* (Jacoby and Dallas, 1981; Jacoby, 1983; Whittlesea et al., 1990; Whittlesea, 1993). Fluency refers to the subjective ease with which a stimulus is being processed, and has been shown to influence many types of cognitive phenomena, including perceptual coherence, intuition, and recognition-memory decisions (e.g., Rajaram, 1993; Kinoshita, 1997; Brown and Marsh, 2009; Topolinski and Strack, 2009, 2010; Verde et al., 2010). In perhaps the best-known illustration of this effect in the memory literature, manipulations that increase the processing fluency of the recognition-memory cue have been found to generate increased feelings of familiarity for this cue (e.g., Jacoby and Whitehouse, 1989; Whittlesea, 1993; Topolinski, 2012). Jacoby and Whitehouse (1989) manipulated the processing fluency of words in a standard recognition memory paradigm by briefly presenting either the same or a different word prior to the test word (e.g., dog–dog, house-dog). The duration of the prime words was manipulated such that they appeared either for 50 or 200 ms. The matching as compared to non-matching prime words were found to increase participants' tendency to call words “old,” which occurred for truly old words and even when they were novel lures that had not been encountered in the study phase. Critically, however, this effect was observed only when the prime word was presented for 50 ms and participants reported that they had been unaware of its presence. Other evidence for fluency-related effects on recognition memory comes from research, for example, in which the blocking of oral movements, by way of chewing gum, specifically impaired familiarity but not recollection-based recognition of words (Topolinski, 2012).

According to attribution models of familiarity, the influence of fluency on familiarity-based memory decisions reflects the operation of a heuristic in which people attribute the subjective ease of processing to a prior encounter with the stimulus in question, in particular when they cannot explain that fluency in other ways (Jacoby and Dallas, 1981; Jacoby, 1983; Whittlesea et al., 1990; Whittlesea, 1993). Evidence in support of the idea that this mechanism plays a role that is specific to familiarity comes from a study by Rajaram (1993) who employed a variant of the Jacoby–Whitehouse paradigm in combination with the Remember-Know (RK) procedure (Tulving, 1985). In this procedure, participants are asked to introspect as to whether recognition of prior occurrence was accompanied by the recovery of pertinent episodic contextual detail in the study phase (“remember”), or whether it was lacking any such detail (“know”). As in Jacoby and Whitehouse's (1989) original study, participants were more likely to endorse test items primed by a matching word as “old.” Critically, this response pattern was observed only for recognition judgments accompanied by a “Know” response, suggesting a specific role of fluency in familiarity-based memory decisions. Although this basic finding has been replicated a number of times, some evidence suggests that this process specificity may only be observed when familiarity and recollection are probed with the binary RK-procedure, but not when probed with two separate quantitative ratings (Higham and Vokey, 2004; Kurilla and Westermann, 2008; Brown and Bodner, 2011).

While memory researchers have focused on the consequence of prior occurrence on recognition-memory judgments, there is also a wealth of studies indicating that the exposure of a stimulus can have affective consequences. Specifically, repeated exposure to various types of stimuli has been reported to increase positive affect toward those stimuli (Zajonc, 1968; Bornstein, 1989; Harmon-Jones and Allen, 2001; Winkielman and Cacioppo, 2001), an observation termed the *mere exposure effect*. For example, prior encounters with a person have been shown to increase positive attitudes (i.e., likeability) and boost perceived attractiveness toward this individual (Moreland and Beach, 1992). Other evidence suggests that influences between prior exposure and positive affect are bi-directional. Monin (2003) found that participants were more likely to judge attractive faces as familiar compared to faces of average attractiveness. Furthermore, familiarity for moderately attractive faces was enhanced when participants had previously made similar judgments on a set of less attractive faces; the sequential contrast presumably led to an increase in perceived attractiveness for the moderately attractive faces, and in turn enhanced feelings of familiarity through the corresponding increase in positive affect (Garcia-Marques et al., 2004; see also Claypool et al., 2008). Other evidence in support of the notion that positive affect can influence recognition judgments comes from research in which participants were asked to generate facial expressions and postures that were consistent either with positive or negative affect, while making recognition memory judgments (Phaf and Rotteveele, 2005; Verde et al., 2010). In one such study, participants in a happy-expression condition judged words with higher confidence as old than participants in a sad-expression condition, leading to a significantly more lenient response bias as revealed in Receiver Operating Characteristics (ROC)-based analyses derived from signal-detection theory (Verde et al., 2010). The authors suggested that this shift in response bias may in fact reflect the strongest type of support for an attributional account of familiarity, as it points to an influence that is, by definition, independent of mnemonic evidence. However, while this and the previously reviewed findings provide evidence that positive affect influences recognition-memory judgments, it is currently not clear whether the influence applies to familiarity assessment as conceptualized and measured in models of recognition memory, which distinguish this process from recollection (Yonelinas, 2001). As a consequence, it also remains unknown whether positive affect shows the same specificity in its influence on recognition-memory judgments as fluency.

A proposal that deserves specific consideration in this context is the notion that positive affect and fluency may be directly linked in their influence on recognition decisions and in other cognitive domains (e.g., stimulus coherence, Topolinski and Strack, 2009); some have in fact argued that processing fluency in itself is hedonically marked (Reber et al., 1998; Harmon-Jones and Allen, 2001; Winkielman and Cacioppo, 2001; Zajonc, 2001; Winkielman et al., 2003). Zajonc (2001) suggested that familiar objects or familiar people are less likely to be dangerous than their unfamiliar counterparts; the positive affective marking of fluency could rapidly signal this safety even in the absence of any detailed analysis of the present situation (see Song and Schwarz, 2009, for related ideas). Some of the strongest support for a direct link between fluency and positive affect comes from psychophysiological research, showing that perceptual priming of visual stimuli not only leads to an increase in subjective liking, but also to the immediate expression of a smile response as measured with electromyographic recordings (Harmon-Jones and Allen, 2001; Winkielman and Cacioppo, 2001).

Recall that processing fluency has been demonstrated to influence familiarity selectively when the latter is probed with binary RK judgments in a recognition-memory task (Rajaram, 1993; Kurilla and Westermann, 2008; Brown and Bodner, 2011). If positive affect is indeed the critical cue that signals processing fluency in familiarity assessment, then it can be predicted that the effects of positive affect should mirror those produced by processing fluency in familiarity-based recognition-memory decisions. In the two experiments reported here, we examined this issue using variants of the priming procedure introduced by Jacoby and Whitehouse (1989). In Experiment 1, we show that the classic effect of processing fluency on recognition memory extends to face stimuli; we report that the masked presentation of a face with the same identity as a subsequent memory probe leads to a liberal shift in response bias in familiarity-based memory decisions; in line with Jacoby and Whitehouse's original findings, this effect is dependent on lack of awareness of the priming manipulation. In Experiment 2, we manipulated affect by introducing primes that were always of a different identity than the subsequent memory probes, but that carried a positive or neutral facial expression; we show that priming with a happy expression produces the same pattern of effects on recognition-memory decisions as the manipulation of fluency in Experiment 1.

## Experiment 1

### Methods

#### Participants

Forty-one participants participated in the study (25 females; Age *M* = 22.3, *SD* = 2.9). All participants gave their written informed consent before participation. One participant was excluded from analyses due to an excessively high level of prime awareness in the third phase of the experiment (discrimination >2 *SD*s above Mean). Another participant was excluded due to an endorsement of less than three “Remember” responses overall, which did not allow us to confirm that the participant understood the RK distinction. All participants were compensated financially, or received course credit, for their participation. The study protocol was approved by a Research Ethics Board at the University of Western Ontario.

#### Stimuli

The stimuli presented were colored images of faces taken from the Karolinska Directed Emotional Faces database (KDEF) as well as the NimStim Emotional Face Stimuli database (Lundqvist et al., 1998; Tottenham et al., 2009). All faces were cropped down to a specific oval template, including the forehead, eyes, nose, mouth, and full jaw, while leaving out hair, jewelry, and ears. This was done to create a more homogenous sample of faces and to reduce large variations in hair style and other stylistic qualities across databases. All face stimuli were surrounded by a rectangular background of Gaussian noise. Overall, 152 neutral faces were used, split into 8 unique sets with 19 faces per set.

Different sets of faces were used as targets (old test items) and novel lures. For each set of old and new items, 3 priming conditions were introduced that corresponded to, (i) Match primes, (ii) Mismatch primes, and (iii) Scrambled primes consisting of ovals with no discernible identity (i.e., a scrambled face oval placed where a face prime would usually be). Having three prime types for both old and new items resulted in a 2 (test status: Old or New) × 3 (prime condition: Match, Mismatch, Scrambled) within-subjects experimental design. Six of the eight sets of 19 faces were used as target faces because both old and new items had three prime types as stated above. One set of 19 faces had to be used as novel non-identity primes for old items, and the final set of 19 unique faces consisted of the novel non-identity primes for the new test items. A complete 8-list counterbalance scheme was created with this grouping, having each set of 19 faces once in all 8 positions.

For the forced-choice prime discrimination task, a pseudo random sample of 20 Mismatch-primed items (half target and lures), and 20 Match-primed items (half targets and lures) were selected. These items were randomized to create the task list. All 8 counterbalancing versions of the experiment had a unique, list-specific, arrangement of items for the discrimination task.

#### Procedure

**Figure 1:**
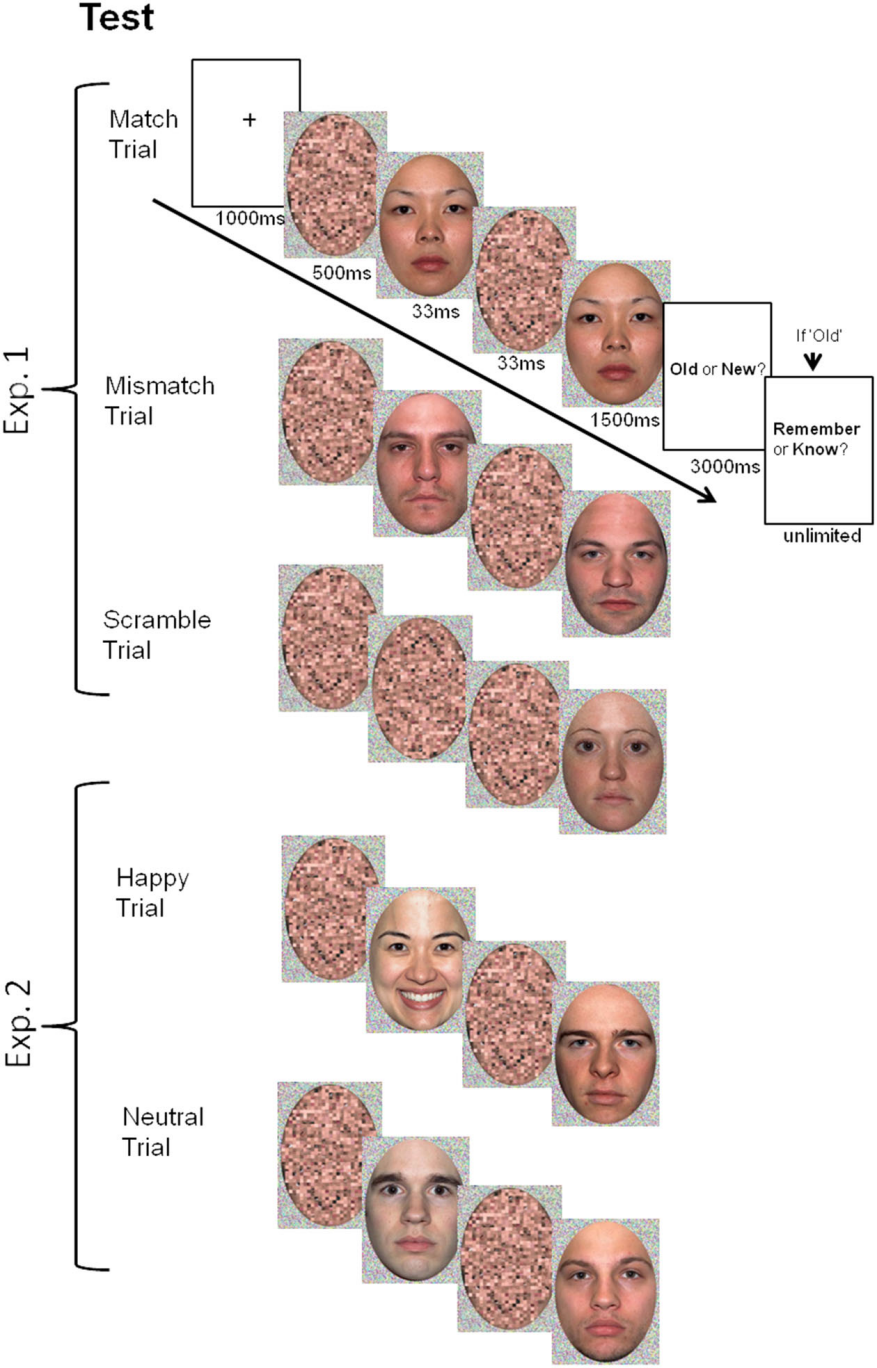
**Illustration of event sequences and trial types for each experiment**.

Figure 1 illustrates the experimental procedure and event sequence used in Experiment 1. Images were presented on a CRT monitor with E-Prime 1.0 (Psychology Software Tools). Participants viewed images at a distance of 50 cm. Faces and their background frames subtended approximately 17° of visual angle.

Prior to the initial study phase, participants were informed that they would be required to make judgments about faces, followed by a memory test. At study, participants were asked to make likeability judgments. A fixation cross appeared for 1000 ms prior to each face, which was then presented for 1500 ms. Immediately after the face disappeared, participants were instructed to judge how much they liked that face using a 6 point scale, with 1 corresponding to “strongly dislike,” and 6 corresponding to “strongly like.” This judgment of likeability was intended to encourage participants to attend to each face during study. After each rating was made, there was a 1000 ms inter-stimulus interval (ISI) prior to the next face presentation. The faces designated to each prime condition were randomized.

After the completion of the study phase, participants received instructions for the recognition test. Overall, test instructions spanned roughly 5 min. Participants were informed that they would then undergo a recognition memory test for the faces presented during the study phase. They were informed that half of the faces in the upcoming test were faces seen during the study phase, and the other half were new faces (i.e., not previously encountered), and also, that the order was random.

At test, each face was preceded by a fixation cross for 1000 ms, immediately followed by a scrambled face oval for 500 ms, serving as the forward mask, which was followed by the prime face. The prime was presented for 33 ms, followed by a 33 ms backward mask, which consisted of the same scrambled face used as the forward mask. Finally, the probe face was presented for 1500 ms (see Figure 1 for schematic representation of trial structure). Participants were not informed about the presence of the prime face, and reported only seeing one continuous scrambled oval prior to each target face. When the probe face disappeared, participants were prompted with “Old” or “New” cues, and were given 3000 ms to make their memory decision. If participants claimed that they recognized the face as “old,” an RK judgment was required in a self-paced manner. Participants indicated whether the experience of recognition was characterized merely by a sense of familiarity for the face in question, or by recollection of episodic detail concerning the initial encounter. Instructions for RK judgments were modeled after those provided by Rajaram (1993). It was emphasized that a single contextual detail would be sufficient to warrant a “Remember” response. To ensure that participants completely understood the phenomenological distinction between familiarity and recollection, they were told to verbally justify their choice for their first two “Remember” and “Know” responses to the experimenter. If the justifications suggested that the distinction was not properly employed, participants obtained feedback with a reiteration of the critical instructions from the experimenter.

After the recognition-memory test, participants were fully debriefed concerning the nature of the priming manipulation during the memory experiment. A prime discrimination task was then administered, which required participants to judge whether the purportedly subliminal prime stimulus matched or mismatched the identity of the probe face. As the primary contrast of interest concerned matching vs. mismatching identity primes, we considered discrimination accuracy in this task as an objective measure of prime awareness. Participants were told that half of the faces were primed by the same face as the probe, and the other half by novel faces. All image presentation parameters were the same as for the recognition memory test. After the probe face disappeared in any given trial, participants were prompted with the text, “Was the prime identity the same as the probe identity?” and were required to provide a “yes” or “no” response.

### Results and discussion

#### Recognition memory in entire sample

A One-Way, repeated measures ANOVA^1^ revealed that overall recognition performance (i.e., discrimination between old and new test faces), as reflected in *d*′ scores, was not significantly affected by the priming manipulations [*F*_(2, 76)_ = 0.393, *p* = 0.642; see Table 1]. Similarly, the accuracy of “Remember” responses was also unaffected by priming [*F*_(2, 76)_ = 0.359, *p* = 0.644]. To examine the influence of the priming manipulation on familiarity in a model that assumes independence between both processes, we first applied the correction procedure introduced by Yonelinas (1999, 2002). Using the correction, we found that familiarity-based discrimination (*d*′) did not differ between prime conditions [*F*_(2, 76)_ = 0.142, *p* = 0.823]. We next examined whether there was an effect on the general bias to endorse faces as “old” when memory decisions were familiarity-based. A measure of response bias (*C-*criterion placement; Macmillan and Creelman, 2005) based on the corrected “Know” responses revealed no shift in response bias [*F*_(2, 76)_ = 0.845, *p* = 0.428]. When we conducted these statistical tests on familiarity-based measures within a model in which no assumption about independence is made (i.e., when no correction is applied), the same pattern of results emerged; there was no evidence for an effect of our experimental fluency manipulation (all *p* > 0.05).

**Table 1:** **Recognition accuracy, familiarity, recollection, and criterion placement for participants with high or low level of prime awareness**.

**Condition-Group**	***d*′ Recognition**	***d*′ Familiarity**	**Recollection**	***C* “Remember”**	***C* Familiarity**
Match-Low	0.74(0.06)	0.62(0.08)	0.13(0.02)	1.49(0.08)	0.53(0.04)
Mismatch-Low	0.75(0.11)	0.60(0.12)	0.13(0.02)	1.38(0.09)	0.70(0.07)
Scramble-Low	0.74(0.12)	0.62(0.11)	0.13(0.03)	1.38(0.09)	0.66(0.05)
Match-High	0.93(0.09)	0.77(0.13)	0.18(0.01)	1.30(0.07)	0.76(0.06)
Mismatch-High	0.97(0.12)	0.83(0.15)	0.17(0.01)	1.32(0.08)	0.73(0.06)
Scramble-High	0.79(0.09)	0.68(0.08)	0.15(0.01)	1.36(0.06)	0.60(0.05)

*High and Low prime awareness groups defined by cut-off at d′ 0.19 prime awareness in the last phase of experiment. “Recollection” refers to “Remember” hits-false alarm rate. SEM in parentheses*.

#### Recognition memory in relation to prime awareness

**Figure 2:**
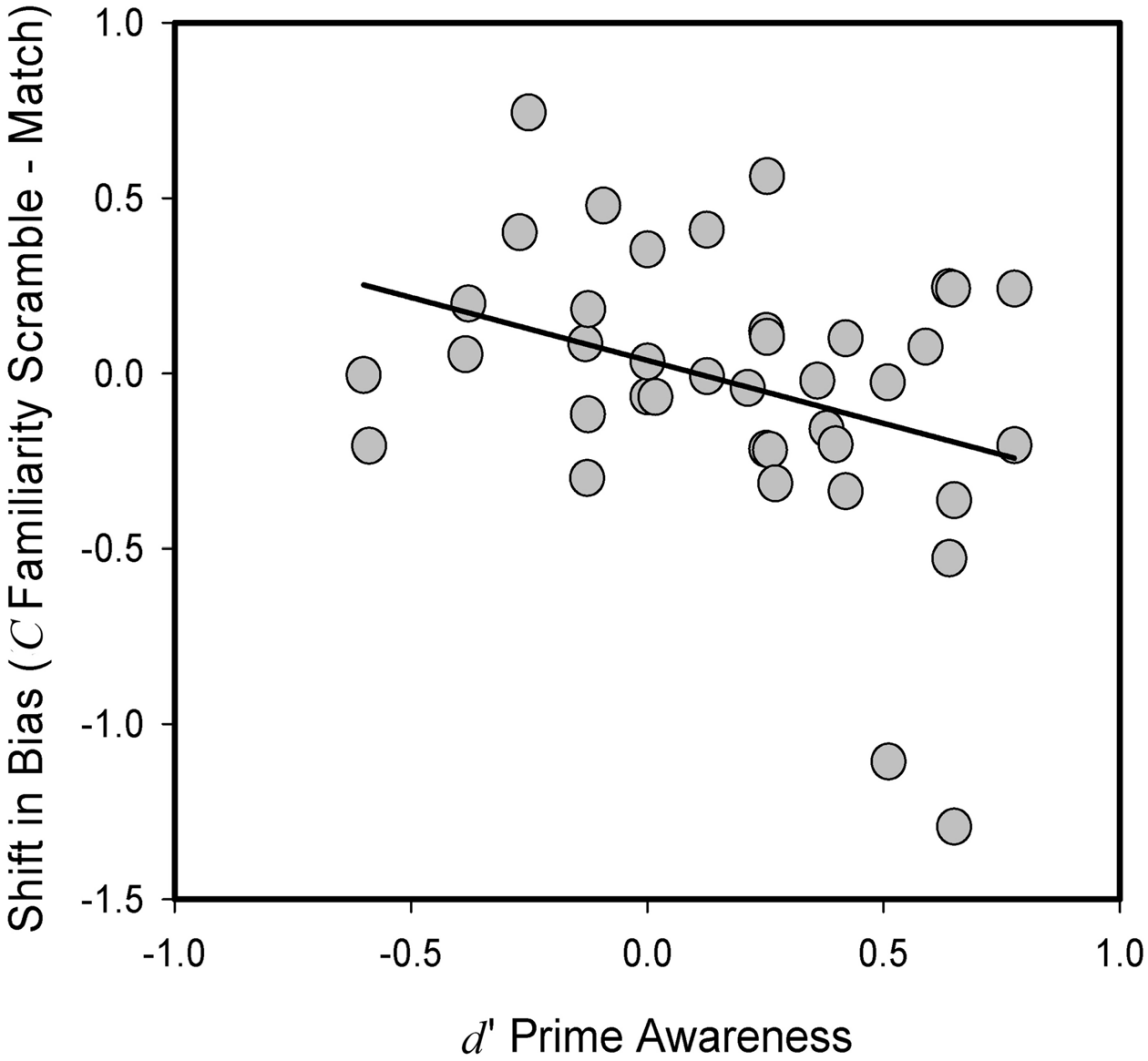
**Relationship between prime awareness (*d*′), as measured in prime-discrimination task, and difference in criterion location (*C*) between Match and Scramble trials for “Know” responses in recognition decisions in Experiment 1**. Positive priming values reflect a more liberal criterion placement for Match relative to Scramble prime condition. Dots represent data of individual participants.

Critically, in a number of prior investigations, fluency effects have only been shown to be present when the manipulation was reported to be subliminal (Jacoby and Whitehouse, 1989; Goldinger and Hansen, 2005; Phaf and Rotteveele, 2005). Therefore, we next examined whether an effect of our fluency manipulation could be revealed if inter-individual differences in prime awareness were taken into account. We estimated prime awareness based on discrimination performance in phase 3 of the experiment. We examined the relationship between the effects of our priming manipulation and prime awareness based on correlational analyses conducted in the entire sample of participants. When we calculated the difference score in criterion placement *C* for corrected familiarity-based responses for the Match as compared to Mismatch priming conditions, we observed no significant relationship to prime awareness scores [*r*_(37)_ = −0.23, *p* = 0.17]. However, a corresponding analysis that employed the other baseline condition, i.e., the presentation of scrambled faces, did reveal a significant relationship; specifically, it revealed a significant negative correlation between the difference scores in criterion placement for corrected familiarity-based responses and prime awareness in phase 3 [*r*_(37)_ = −0.34, *p* < 0.05; see Figure 2]. This negative correlation indicates that those participants who were less aware of the priming manipulation tended to exhibit a shift toward a more liberal response bias for familiarity-based decisions in the experimental condition that aimed to increase processing fluency. Inasmuch as any two faces have a high degree of perceptual overlap, the presentation of scrambled faces is arguably the more appropriate baseline condition in the current experiment to reveal effects of perceptual fluency. We note that the negative correlation between response bias for familiarity-based memory decisions and prime awareness also emerged when criterion placement was calculated without correction, i.e., within a model that does not assume independence between familiarity and recollection [*r*_(37)_ = −0.33, *p* < 0.05]. By contrast, an examination of response bias *C* for recollection-based “Remember” responses did not reveal any relationship between any effects of the priming manipulation and prime awareness [*r*_(37)_ = 0.16, *p* = 0.340], in line with the negative findings obtained in the analysis that was restricted to participants with low prime awareness.

We also conducted similar analyses restricted to a subsample of participants who clearly showed no evidence for prime awareness in phase 3. All planned comparisons are two-tailed unless otherwise noted. Overall, participants' *d*′ scores were significantly above chance as measured by a one-sample *t*-test against 0 [*t*_(38)_ = 3.145, *p* < 0.01], suggesting that for the sample as a whole priming could not be assumed to be subliminal. While the average *d*′ score in the prime-discrimination task was 0.19 (*SD* = 0.37), there were considerable inter-individual differences that were reflected in a range of scores between −0.60 and 0.78. These inter-individual differences allowed us to examine whether our fluency manipulation had any effect on recognition decisions that was dependent on absence of prime awareness. Using the Mean of *d*′ in the prime-discrimination task (*M* = 0.19) as the cut off score, we identified a subsample of participants (*n* = 17) in whom prime discriminability was clearly not above chance level, as confirmed statistically with a one-sample *t*-test against 0 [in fact, performance was statistically below chance level in this sample; *t*_(16)_ = 3.095, *p* < 0.01]. To determine whether the priming manipulation had the predicted effect on response bias in this subsample, we examined a planned contrast on *C*-values for corrected familiarity responses between the Match relative to both the Mismatch and Scramble priming conditions; this test revealed a more liberal response bias for familiarity-based memory decisions in the Match condition as predicted [*F*_(1, 16)_ = 4.916, *p* < 0.05 (one-tailed)]. The effect was also present when we calculated response bias with uncorrected familiarity scores [*F*_(1, 16)_ = 7.09, *p* < 0.05]. Critically, we observed no differences in familiarity-based discrimination (*d*′) across priming conditions regardless of whether it was calculated with or without the independence assumption (*p*'s > 0.8). Also, a corresponding test on response bias (*C*) for recollection-based responses did not reveal any effect of priming [*F*_(1, 16)_ = 0.766, *p* = 0.394].

Finally, a comparison of the correlation between prime awareness and the effects of priming on the different memory-performance measures revealed a significantly larger correlation for *C* familiarity than *d*′ familiarity [*t*_(36)_ = 1.695, *p* < 0.05 (one-tailed)] and recollection [*t*_(36)_ = 2.239, *p* < 0.05], using Hotelling's *t*-test, further highlighting the specificity of the priming effect.

Overall, Experiment 1 demonstrated that primes that matched the identity of the subsequent target faces selectively boosted their perceived familiarity; this effect was only observed in participants who were unaware of the presence of the prime stimuli. These results show that the classic fluency effect first described by Jacoby and Whitehouse (1989) for verbal stimuli extends to recognition-memory for faces. Critically, they also show that the effect operates with the same specificity on binary RK judgments as described in past research with verbal stimuli (Rajaram, 1993; Higham and Vokey, 2004; Kurilla and Westermann, 2008; Brown and Bodner, 2011). In Experiment 2, we sought to build on the findings of Experiment 1 and examine the role of positive affect. Given the proposed link between fluency, positive affect, and feelings of familiarity (Harmon-Jones and Allen, 2001; Monin, 2003; Garcia-Marques et al., 2004; Phaf and Rotteveele, 2005), we asked whether a priming-based manipulation of positive affect would produce the same effect on familiarity-based memory decisions as the manipulation of fluency in Experiment 1, and whether any such effect would also show the same relationship to prime awareness. We again focused on recognition memory for faces and employed a priming procedure similar to that in Experiment 1, with the exception that the prime stimuli consisted of happy (i.e., smiling) faces conveying positive affect, or neutral faces as a control condition. To control for any fluency effects based on perceptual overlap between primes and targets across experimental conditions, the faces employed as primes were always of a different identity than the faces subsequently presented for memory judgments. We expected that the presentation of happy as compared to neutral face primes would result in a shift toward a more liberal response bias for familiarity-based memory decisions, specifically in those participants who were unaware of the priming manipulation.

## Experiment 2

### Methods

#### Participants

Seventy-two individuals who gave written informed consent participated in the study for compensation (Age *M* = 21.1, *SD* = 2.5, 45 females). Data from five participants were excluded from analyses due to exceptionally high prime awareness (discrimination scores > two *SD* above Mean; see Hannula et al., 2005, for rationale). Data from seven participants were excluded from analyses given that they had chance recognition performance or did not provide at least three “Remember” responses.

#### Stimuli

The images of faces used in this experiment were prepared in an identical manner as those in Experiment 1 (i.e., cropped face and noise frame). Additional faces from the Radboud Face Database were included in the set so as to increase the numbers of items available as primes with expression of positive or neutral affect (Langner et al., 2010). The final set consisted of 144 face identities; for each identity there was a version with a happy and a neutral facial expression. The entire set was divided into four lists, with two lists serving as targets and two lists serving as lures in the recognition-memory test. Old and new test items were primed by either a happy (list 1) or a neutral face (list 2), leading to a 2 (test status: Old or New) × 2 (prime condition: Happy, Neutral) experimental design. Assignment of lists to conditions was counterbalanced across participants. All face probes presented for memory judgments were shown with neutral facial expression. Each probe face was paired with a prime of a different identity to avoid any influence of identity-based priming (i.e., as manipulated in Experiment 1). Each identity was used only once as prime (i.e., primes were trial unique). However, given the limited set of 144 identities available for the entire experiment, each prime had the same identity as one of the faces used as memory probes on other trials in the same experimental condition. Critically, identities presented as primes appeared equally often before and after their use as probes across experimental conditions.

#### Procedure

The procedure for the study phase was identical to Experiment 1, except that faces were presented at a duration of 500 ms rather than 1500 ms. After a 5-min delay, participants performed the recognition-memory test. The procedure and presentation parameters for this test were the same as in Experiment 1, including facial primes being presented for 33 ms, except for a difference in the duration of the presentation of probe faces; faces that were to be judged as “old” or “new” were presented for 250 ms rather than 1500 ms. After the recognition-memory test, participants were probed for prime awareness and were fully debriefed concerning the nature of the priming manipulation in the memory experiment. Subsequently, they were probed for prime awareness with a task that had the same structure as the memory test but required participants to judge whether the purportedly subliminal prime on every trial had a happy or a neutral facial expression. Participants were told that half of the probes were primed by happy faces, and the other half by neutral faces. Stimuli and all image presentation parameters were the same as used in the recognition-memory test. Participants were required to indicate the perceived expression with a button press.

### Results

#### Recognition memory in entire sample

A paired *t*-test showed that overall recognition performance, as reflected in *d*′ scores, was not significantly affected by the priming manipulation [*t*_(59)_ = 1.220, *p* = 0.227]. Similarly, the accuracy of “Remember” responses was unaffected by this manipulation [*t*_(59)_ = 0.771, *p* = 0.444]. An examination of familiarity-based discrimination, calculated as *d*′ based on corrected “Know” responses or as *d*′-based on uncorrected “Know” responses, did not reveal any differences between the priming conditions [*t*_(59)_ = 1.063, *p* = 0.292; *t*_(59)_ = 1.03, *p* = 0.307]. Finally, there was also no difference in response bias for familiarity-based responses across experimental conditions, regardless of whether *C* was calculated with [*t*_(59)_ = 0.720, *p* = 0.474] or without [*t*_(59)_ = 0.773, *p* = 0.443] correction for independence.

#### Recognition memory in relation to prime awareness

**Figure 3:**
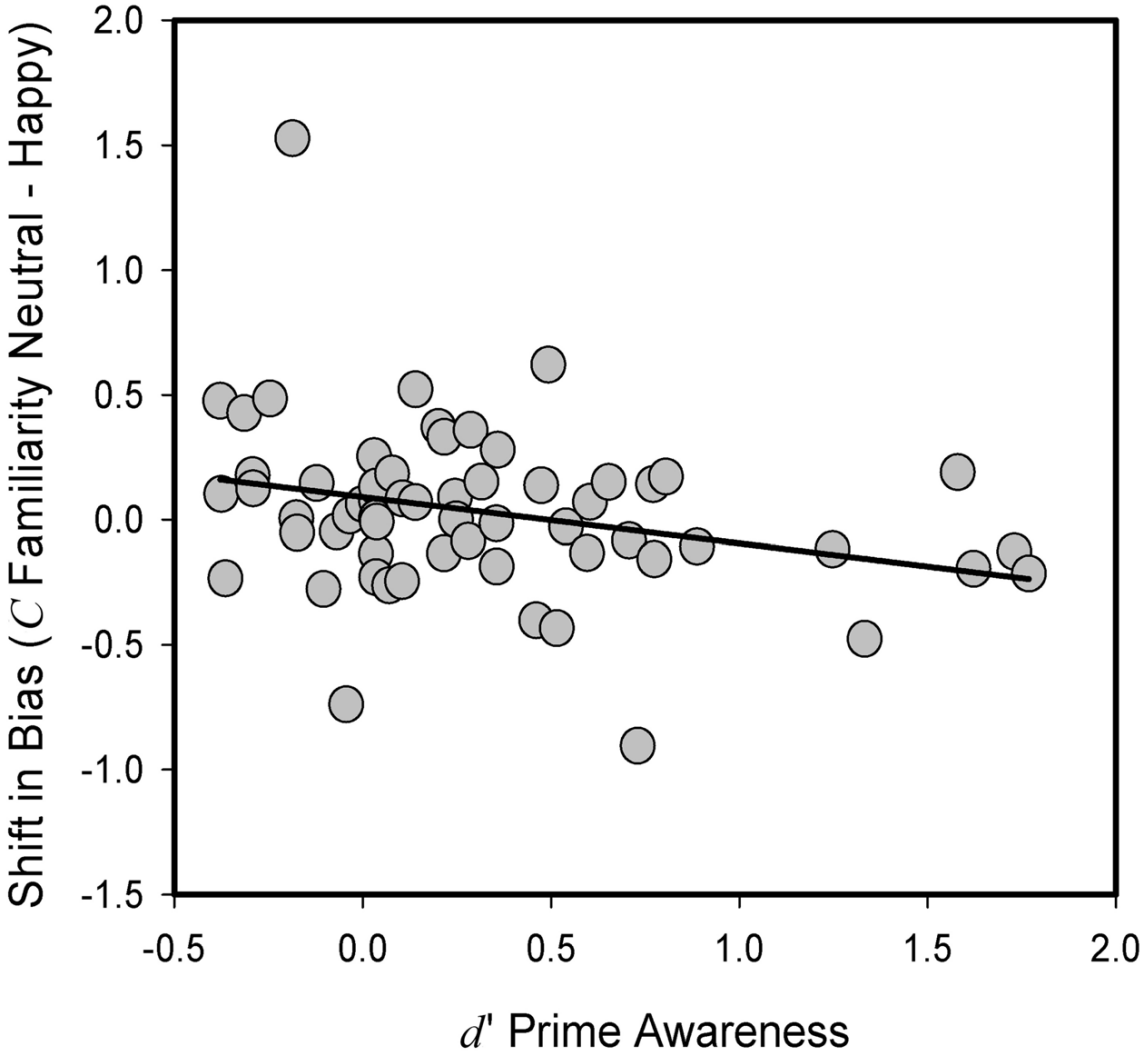
**Relationship between prime awareness (*d*′), as measured in prime-discrimination task, and difference in criterion location (*C*) between Happy and Neutral trials for “Know” responses in recognition decisions in Experiment 2**. Positive priming values reflect a more liberal criterion placement for Happy relative to Neutral prime condition. Dots represent data of individual participants.

As in Experiment 1, we next examined whether an effect of our experimental manipulation could be revealed if inter-individual differences in prime awareness, as measured in the prime discrimination task of the experiment, were taken into account. Prime awareness scores were on average above 0 (Mean *d*′ = 0.32, *SD* = 0.53) and ranged between −0.38 and 1.77; a one-sample *t-test* confirmed that values were significantly above chance [*t*_(59)_ = 4.665, *p* < 0.0001], suggesting that prime awareness could not be assumed to be subliminal in the entire sample. To determine whether there was any effect of our priming manipulation on response bias in familiarity-based memory decisions that might depend on levels of prime awareness, we again calculated a difference score for *C* across the two priming conditions in each participant and examined the relationship between these scores and scores of prime awareness, as reflected in *d*′ of the prime discrimination task. Here, a significant negative correlation emerged, regardless of whether *C* was calculated within a model that assumed independence between familiarity and recollection [*r*_(58)_ = −0.285, *p* < 0.05; see Figure 3], or within a model that did not assume independence [*r*_(58)_ = −0.25, *p* < 0.05, one-tailed]. Mirroring the pattern we observed in Experiment 1, as prime awareness decreased, there was a shift toward a more liberal response bias in familiarity-based decisions for stimuli primed with happy as compared to neutral faces. In other words, to the extent that participants had low awareness of the priming manipulation, the presentation of positive, affective primes increased the likelihood that they would endorse any probe face as familiar. Critically, we observed no corresponding relationship to familiarity-based discrimination, regardless of whether the latter was calculated with or without correction for independence [*r*_(58)_ = −0.05, *p* = 0.683; *r*_(58)_ = −0.05, *p* = 0.702]. Also, this effect was specific to familiarity-based decisions as we did not observe any significant relationship when we focused on the response bias for “Remember” responses [*r*_(58)_ = −0.08, *p* = 0.564].

As in Experiment1, we also conducted similar analyses restricted to a subsample of participants who clearly showed no evidence for prime awareness in phase 3. A cut-off at the Mean of prime-discrimination (*d*′ = 0.32) allowed us to select such a group of participants (*n* = 37) who did not show above chance levels of prime awareness, as confirmed with a one-sample *t*-test of the *d*′ discrimination scores against a population mean of 0 [*t*_(36)_ = 0.238, *p* = 0.813]. As predicted, memory judgments on items that were primed with happy faces were characterized by a significantly more liberal response bias in familiarity-based decisions; this was the case when the independence correction was applied [*t*_(36)_ = 1.746, *p* < 0.05 (one-tailed)], and a significant trend emerged when uncorrected [*t*_(36)_ = 1.671, *p* < 0.0515 (one-tailed)]. By contrast, there were no differences in familiarity-based discrimination (*d*′) (all *p* > 0.64 with or without correction for independence; see Table 2). Also, there was no shift in bias for “Remember” responses [*t*_(36)_ = 0.380, *p* = 0.706], highlighting again the specificity of the effect for familiarity that we observed.

**Table 2:** **Recognition accuracy, familiarity, recollection, and criterion placement for participants with high or low level of prime awareness**.

**Condition-Group**	***d*′ Recognition**	***d*′ Familiarity**	**Recollection**	***C* “Remember”**	***C* Familiarity**
Happy-Low	0.45(0.03)	0.34(0.04)	0.08(0.01)	1.25(0.04)	0.54(0.03)
Neutral-Low	0.43(0.03)	0.31(0.04)	0.10(0.01)	1.22(0.04)	0.64(0.03)
Happy-High	0.58(0.04)	0.43(0.05)	0.11(0.01)	1.13(0.03)	0.54(0.03)
Neutral-High	0.46(0.04)	0.30(0.05)	0.11(0.01)	1.04(0.03)	0.46(0.03)

*High and Low prime awareness groups defined by cut-off at d ′ 0.32 prime awareness in the last phase of experiment. “Recollection” refers to “Remember” hits-false alarm rate. SEM in parentheses*.

Furthermore, to highlight the specificity of the observed priming effect, we also conducted a 2(prime type) × 2(awareness group) group factorial MANOVA incorporating *C* familiarity, *d*′ familiarity, and recollection estimates, which revealed a significant prime × group interaction [*F*_(3, 56)_ = 3.345, *p* < 0.025]. Three univariate tests were employed to examine this effect on *C* familiarity, *d*′ familiarity, and recollection estimates individually. They revealed a significant prime × group interaction for *C* familiarity [*F*_(1, 58)_ = 5.911, *p* < 0.018], with no other significant effects.

## General discussion

The present experiments aimed to compare the effects of perceptual fluency and positive affect on familiarity assessment for faces with neutral expressions. Using variants of the Jacoby–Whitehouse paradigm in combination with the RK procedure, we found that the manipulation of positive affect in Experiment 2 produced a pattern of results that closely mirrored the one produced by the classic manipulation of processing fluency in Experiment 1. In both experiments, the critical effect was specific to familiarity-based responses. Moreover, in both experiments it was reflected in a shift toward a more liberal response bias, rather than in changed discrimination. Finally, in both experiments, the effect was found to be related to prime awareness; it was present only in participants who reported a lack of such awareness on a separate prime-discrimination task. These findings add to a growing body of evidence that points not only to a role of fluency, but also of positive affect in familiarity assessment. As such they are consistent with the idea that fluency itself may be hedonically marked.

### Perceptual fluency and enhanced face familiarity

Experiment 1 demonstrated that the Jacoby–Whitehouse effect can be observed in recognition memory decisions for faces, and is reflected in a more liberal response bias in familiarity-based recognition decisions for such stimuli. To our knowledge, this effect has previously not been demonstrated with faces. At the same time, these results extend an existing literature on the role of perceptual fluency in memory judgments for faces with other types of fluency manipulations. For example, in prior studies it has been demonstrated that an enhancement of the perceptual clarity of test faces increases participants' tendency to judge them as “old” (Whittlesea et al., 1990; Kleider and Goldinger, 2004). Using the RK procedure, we observed that such a shift in response bias, at least within the Jacoby–Whitehouse paradigm, is specific to familiarity as defined by dual-process models of recognition memory (Yonelinas, 2001). Past studies investigating the role of fluency in face recognition have not explicitly examined the influence on both processes, sometimes simply assuming that familiarity was specifically affected in the critical memory decisions (e.g., Monin, 2003). However, this is not a trivial point; recent studies have shown that, under some circumstances, fluency manipulations can also influence recollection. Evidence from a number of studies, with stimuli other than faces, suggests that selective effects of fluency on familiarity may only be observed when familiarity and recollection are probed with the binary RK-procedure, but not when probed with two separate quantitative ratings (Higham and Vokey, 2004; Kurilla and Westermann, 2008; Brown and Bodner, 2011). While measurement issues remain a topic of active research and strong disagreement in the dual-process literature (e.g., Dunn, 2008; Wixted and Mickes, 2010; Yonelinas et al., 2010; Migo et al., 2012) it should be noted that even when both processes have been probed with two separate ratings, the classic Jacoby–Whitehouse effect has consistently been observed for familiarity-based responses. One challenge in such experiments is that participants may find it difficult, if not impossible, to separate both memory dimensions. In an experiment in which a specific effort was made to minimize contamination of memory judgments across the two types of ratings (by obtaining them in two independent samples of participants), it was found that the effect was still larger for familiarity than for recollection (Brown and Bodner, 2011).

Another issue to consider when evaluating the process-specificity of fluency effects is that multiple sources of fluency may contribute to familiarity. In research with verbal stimuli that have semantic meaning, it has long been recognized that familiarity may not only be linked to the fluency of perceptual processing but also of conceptual processing (see Dew and Cabeza, 2011 and Voss et al., 2012, for reviews). For example, masked priming effects on recognition-memory decisions have also been found with primes that were conceptually related, rather than identical, to the subsequent memory probes (Rajaram and Geraci, 2000; Taylor and Henson, 2012; Dew and Cabeza, 2013). Such conceptually-based priming has recently been shown to influence recollection estimates even in the context of binary R/K judgments (Taylor and Henson, 2012; Taylor et al., 2013). It is possible that the direct repetition of a meaningful verbal stimulus in the Jacoby and Whitehouse paradigm also induces fluency effects at the level of conceptual processing. To the extent that, in the present study, we observed the effect with novel faces that could not have been encountered prior to the experiment, and that would have no direct meaning attached, we arguably reduced any potential influence of conceptual fluency. This interpretation is in line with ERP research showing that familiarity for faces previously encountered only in the experimental context (i.e., without pre-existing meaning) has an electrophysiological signature that is distinct from the one typically observed in association with conceptual priming or conceptually-based familiarity for verbal stimuli (MacKenzie and Donaldson, 2007; Voss et al., 2012). An interesting question that deserves further direct investigation is whether perceptual fluency is indeed more selective in its effect on familiarity-based recognition memory than conceptual fluency.

### Positive affect and enhanced face familiarity

In Experiment 2 we reasoned that if the impact of fluency in the Jacoby–Whitehouse paradigm is closely tied to feelings of positive affect, then primes with happy facial expression should also lead to an increase in the perceived familiarity of subsequently presented memory probes. Faces with happy as compared to neutral expressions did indeed lead to a liberal shift in familiarity-based response bias, even though the primes were always of a different identity than the subsequently presented memory probes. A particularly noteworthy aspect of these results is that the mnemonic effects of positive affective priming, which have previously been reported for memory decisions on verbal stimuli (Garcia-Marques et al., 2004; Phaf and Rotteveele, 2005), extend to arguably the most relevant social stimulus class—human faces. From an evolutionary perspective, positive affect may serve an adaptive purpose in leading people to construe familiar others as safe, and promote social interaction (Goodman and Leyden, 1991). It is in line with the notion that positive affect may signify safe, non-threatening situations more broadly (Zajonc, 2001; Song and Schwarz, 2009). Interestingly, in past studies it has also been demonstrated that the perceived happiness of faces with ostensibly neutral expression is positively correlated with the perceived trustworthiness of those faces (Winston et al., 2002; Todorov and Duchaine, 2008). In future studies, it will be important to determine whether priming of recognition decisions with negative facial expressions, such as fear or anger, produces effects that differ from those induced by positive affect. Given that, the presentation of fearful faces has been shown to enhance the accuracy of subsequent visual perceptual judgments (Phelps et al., 2006), it is possible that such primes would also improve familiarity-based memory discrimination. Fear may engender a state of sensory hyper-vigilance (Whalen, 1998; Susskind et al., 2008) that actually boosts memory accuracy, rather than induce a shift in response bias.

That we observed a liberal shift in response bias for familiarity-based decisions with a manipulation of positive affect that was independent of the memoranda themselves (i.e., that impacted recognition decisions for neutral faces), is evidence that clearly speaks in favor of an attributional account of the present results. As noted by Verde et al. (2010), interpreting similar effects when the recognition probes themselves evoke positive affect is not straightforward. Indeed, these authors showed that, when the stimulus content is the source of affect, an increase in false alarms for positive stimuli may not reflect a shift in response criterion, but rather a drop in memory accuracy due to higher semantic overlap between these stimuli.

### Familiarity and prime awareness

In both present experiments, effects of priming on familiarity-based memory decisions were observed only in those participants who were unaware of the priming manipulation, as reflected in their performance in our separate prime-discrimination task. Additional correlational analyses also revealed a negative association between the shift in response bias produced by priming and prime-discrimination performance when we examined the entire sample of participants in each experiment. Across participants, lower levels of prime discriminability were associated with an increase in the difference between response criterion for familiarity-based responses for the Match vs. Scramble conditions in Experiment 1, and for happy vs. neutral primes in Experiment 2. The difference in response bias for familiarity-based “Know” responses was such that Match primes and Happy primes were associated with a significantly more liberal response bias than their respective control conditions. That the effectiveness of our experimental manipulation critically depended on lack of prime awareness can be seen as additional support for the notion that familiarity relies, at least in part, on an attribution process that is based on fluency and positive affect. This idea was part of the original account of the Jacoby and Whitehouse effect offered by the authors (Jacoby and Whitehouse, 1989), and was subsequently developed into the Discrepancy Attribution Hypothesis by Whittlesea and Williams (2000, 2001a,b). The latter hypothesis is based on the notion that the critical determinant of such an attribution process is not the absolute level of experienced fluency but the deviation from related expectations; misattributions are most likely to occur when the level of experienced fluency deviates from some expected standard or norm (Whittlesea and Williams, 2001b). From this perspective, the present findings could suggest that the fluency afforded by priming was experienced as surprising or unexpected only in participants who were unaware of the primes, and who attributed it to prior exposure with the test item due to this discrepancy. For those participants who did demonstrate some awareness of the critical prime stimuli, by contrast, any related increase in fluency may not have been experienced as surprising, given that it could be attributed to exposure to the primes. Inasmuch as the same pattern of results emerged for both priming manipulations, our findings suggest that familiarity attributions of positive affect and fluency are governed by the same set of principles, and that unexpected positive affect is attributed to prior experience, even in the absence of a corresponding change in perceptual fluency. As such our findings also provide support for theoretical accounts that identify experienced positive affect as the proximal cue that signals fluency in familiarity-based recognition memory decisions, and in other intuitive judgments (e.g., Reber et al., 1998; Harmon-Jones and Allen, 2001; Monin, 2003; Topolinski and Strack, 2009).

Although the priming-related shift in response bias was critically dependent on participants' awareness of the primes in the present experiments, it is important to note that attributions based on manipulations of fluency do not always require that participants be unaware of the source of this fluency. For example, some studies (Phaf and Rotteveele, 2005; Topolinski and Strack, 2009) have shown that affect-related fluency manipulations (i.e., instructed contraction of zygomatic muscle) of which participants are presumably aware when they follow instructions can also lead to attributional effects similar to those obtained here. Perhaps the key determinant of these effects is not whether participants are aware of the source of fluency itself (i.e., muscle tension), but rather any subjectively perceived or inferred relationship between the fluency manipulation and the task judgments; participants who do not perceive any such relationship would be more likely to use fluency as a cue to inform their judgments. In this context it is worth noting that experiments with affect-related fluency manipulations typically introduce explicit deception so as to hide any potential link whereas in the current experiments this was not necessary. From this perspective, despite the supraliminal nature of some fluency manipulations, the pattern of attributional effects may still be the same and could still be accommodated within the Discrepancy-Attribution framework.

## Conclusions

In the current experiments, we demonstrated that perceptual fluency and positive affect influence recognition memory in a highly similar and specific manner that can well be understood in the context of dual-process models of recognition memory. Although our results support an attributional account of familiarity, we hasten to add that they do not imply that familiarity must always be a product of attribution. Instead, we would argue that familiarity can result from multiple underlying sources, including direct access to pertinent stored memory traces, as modeled in many computational accounts of familiarity assessment (see Clark and Gronlund, 1996, for review), and as assumed in much current cognitive neuroscience research (see Norman, 2010, see Skinner and Fernandes, 2007, for review). An important goal for future research is to shed light on the interplay between attributional mechanisms and direct access to stored representations in familiarity-based memory judgments.

### Conflict of interest statement

The authors declare that the research was conducted in the absence of any commercial or financial relationships that could be construed as a potential conflict of interest.

## Footnotes

^1^Degrees of freedom were adjusted for violations of sphericity using Greenhouse–Geisser epsilon multipliers.
