# Supplementary material for: Parallel effects of processing fluency and positive affect on familiarity-based recognition decisions for faces
Source: Front Psychol. 2014 Apr 22;5:328. doi: 10.3389/fpsyg.2014.00328 (PMC4001004; doi:10.3389/fpsyg.2014.00328)
Supplement: Supplementary file 1 [file DataSheet1.DOCX]

**Appendix A**

Proportion 'Remember', 'Know', and 'New' responses for old faces (studied) and new faces during the recognition test for Experiment 1. Raw proportions are separated by prime awareness group.

**New Faces Old Faces**

**Group R K New R K New**

**Match**-Low 0.07(0.02) 0.20(0.02) 0.74(0.02) 0.19(0.02) 0.34(0.02) 0.47(0.02)

**Mismatch**-Low 0.08(0.02) 0.18(0.02) 0.74(0.03) 0.21(0.02) 0.28(0.02) 0.51(0.02)

**Scramble**-Low 0.08(0.02) 0.17(0.02) 0.75(0.02) 0.20(0.02) 0.30(0.02) 0.49(0.03)

**Match**-High 0.07(0.01) 0.16(0.02) 0.77(0.01) 0.25(0.02) 0.28(0.02) 0.47(0.01)

**Mismatch**-High 0.07(0.01) 0.18(0.02) 0.75(0.02) 0.25(0.01) 0.29(0.02) 0.46(0.01)

**Scramble**-High 0.07(0.01) 0.19(0.01) 0.74(0.02) 0.22(0.01) 0.31(0.02) 0.47(0.02)

**Note:** High and Low prime awareness groups defined by cut-off at *d'* 0.19 prime awareness in the last phase of experiment. SEM in parentheses.
