# Supplementary material for: Parallel effects of processing fluency and positive affect on familiarity-based recognition decisions for faces
Source: Front Psychol. 2014 Apr 22;5:328. doi: 10.3389/fpsyg.2014.00328 (PMC4001004; doi:10.3389/fpsyg.2014.00328)
Supplement: Supplementary file 2 [file DataSheet2.DOCX]

**Appendix B**

Proportion 'Remember', 'Know', and 'New' responses for old faces (studied) and new faces during the recognition test for Experiment 2. Raw proportions are separated by prime awareness group.

**New Faces Old Faces**

**Group R K New R K New**

**Happy**-Low 0.11(0.01) 0.22(0.01) 0.67(0.01) 0.19(0.01) 0.30(0.01) 0.51(0.01)

**Neutral**-Low 0.10(0.01) 0.21(0.01) 0.69(0.01) 0.20(0.01) 0.26(0.01) 0.54(0.01)

**Happy**-High 0.11(0.01) 0.22(0.01) 0.68(0.01) 0.22(0.01) 0.31(0.02) 0.47(0.02)

**Neutral**-High 0.12(0.01) 0.24(0.01) 0.63(0.01) 0.24(0.01) 0.30(0.01) 0.46(0.02)

**Note:** High and Low prime awareness groups defined by cut-off at *d'* 0.32 prime awareness in the last phase of experiment. SEM in parentheses.
